# Diagnostic accuracy of magnetic resonance imaging targeted biopsy techniques compared to transrectal ultrasound guided biopsy of the prostate: a systematic review and meta-analysis

**DOI:** 10.1038/s41391-021-00449-7

**Published:** 2021-09-21

**Authors:** E. J. Bass, A. Pantovic, M. J. Connor, S. Loeb, A. R. Rastinehad, M. Winkler, Rhian Gabe, H. U. Ahmed

**Affiliations:** 1 Imperial Prostate. Division of Surgery, Department of Surgery and Cancer, Faculty of Medicine, Imperial College London, London, UK; 2 Imperial Urology, Division of Cancer, Cardiovascular Medicine and Surgery, Imperial College Healthcare NHS Trust, London, UK; 3 Centre of Research Excellence in Nutrition and Metabolism, Institute for Medical Research –, Belgrade, Serbia; 4 Department of Urology and Population Health, New York University and Manhattan Veterans Affairs, New York, NY USA; 5 Department of Urology, Lenox Hill Hospital at Northwell Health, New York, NY USA; 6 Centre for Cancer Prevention, Wolfson Institute of Preventive Medicine, Queen Mary University of London, London, UK

**Keywords:** Prostate cancer, Prostate cancer, Outcomes research

## Abstract

**Background:**

Multiparametric MRI localizes cancer in the prostate, allowing for MRI guided biopsy (MRI-GB) 43 alongside transrectal ultrasound-guided systematic biopsy (TRUS-GB). Three MRI-GB approaches exist; visual estimation (COG-TB); fusion software-assisted (FUS-TB) and MRI ‘in-bore’ biopsy (IB-TB). It is unknown whether any of these are superior.

We conducted a systematic review and meta-analysis to address three questions. First, whether MRI-GB is superior to TRUS-GB at detecting clinically significant PCa (csPCa). Second, whether MRI-GB is superior to TRUS-GB at avoiding detection of insignificant PCa. Third, whether any MRI-GB strategy is superior at detecting csPCa.

**Methods:**

A systematic literature review from 2015 to 2019 was performed in accordance with the START recommendations. Studies reporting PCa detection rates, employing MRI-GB and TRUS-GB were included and evaluated using the QUADAS-2 checklist. 1553 studies were found, of which 43 were included in the meta-analysis.

**Results:**

For csPCa, MRI-GB was superior in detection to TRUS-GB (0.83 vs. 0.63 [*p* = 0.02]). MRI-GB was superior in detection to TRUS-GB at avoiding detection of insignificant PCa. No MRI-GB technique was superior at detecting csPCa (IB-TB 0.87; COG TB 0.81; FUS-TB 0.81, [*p* = 0.55]). There was significant heterogeneity observed between the included studies.

**Conclusions:**

In patients with suspected PCa on MRI, MRI-GB offers superior rates of csPCa detection and reduces detection of insignificant PCa compared to TRUS-GB. No individual MRI-GB technique was found to be better in csPCa detection. Prospective adequately powered randomized controlled trials are required.

## Introduction

Prostate cancer (PCa) is suspected after either an abnormal digital rectal examination (DRE) or raised prostate-specific antigen (PSA) test, or both. Once suspected, PCa requires tissue confirmation. Traditionally the method to achieve this is the systematic transrectal, ultrasound-guided prostate biopsy (TRUS-GB). The introduction of multiparametric magnetic resonance imaging (mpMRI) allows the identification of discrete areas of abnormal tissue that have a higher likelihood for harbouring clinically significant prostate cancer (csPCa). Thus, this confers a higher sensitivity than TRUS-biopsy alone, approximately 87% to 93%, depending on the definition of csPCa [1]. Current guidance now recommends mpMRI to be carried out prior to the first biopsy [2]. Its use prior to biopsy has grown rapidly, to near 100% in the UK [3]. The ability of mpMRI to localize disease in the prostate allows for targeted or guided biopsy (MRI-GB).

Alongside the proliferation in MRI use pre-biopsy, there is significant interest in MRI-GB [4]. For instance, there was a 20-fold increase in the use of MRI-GB in the United States in recent years [4]. Currently, there is variation in how MRI-GB is performed. Three major technical approaches to MRI-GB are generally employed. First, visual estimation or cognitive targeting (COG-TB) in which a region of interest (ROI) is identified prior to biopsy and the biopsy operator estimates where it might be on an ultrasound image. Second, software-assisted fusion (FUS-TB) involves identifying and contouring ROIs on MR-images before biopsy and overlaying these with the prostate contours on ultrasound images during the biopsy procedure. This can be elastic (deformable registration to reflect deformation of the prostate) or rigid (an overlay of MRI to ultrasound images with some adjustment of rotation). Last, in-bore biopsy (IB-TB) (or in-gantry) involves performing the biopsy in the MRI scanner, guided by MR imaging taken immediately after each needle placement.

There is currently no consensus on whether any of these approaches is superior in terms of cancer detection. Thus, we conducted a systematic review and meta-analysis to address first, whether MRI-GB is superior to TRUS-GB at detecting clinically significant PCa (csPCa). Second, if MRI-GB is better at *not* detecting insignificant PCa, and third if any of the three MRI-GB strategies is superior at detecting csPCa.

## Patients and methods

### Search strategy

The current systematic review and meta-analysis aimed to update the results published by Wegelin et al., 2017 [5] and followed the guidelines suggested by the Preferred Reporting Items for Systematic Reviews and Meta-Analyses statement [6]. We employed the identical search strategy, using the keywords “Prostate OR Prostatic Neoplasm” AND “Biopsy” AND “Magnetic Resonance Imaging OR Image-Guided Biopsy”. The search was performed in PubMed, Embase, and CENTRAL databases with the language restricted to English. As the review by Wegelin et al. [6] included studies published before 15/12/2015, the time frame was restricted to 15/12/2015 to 29/07/2019. The review was registered on PROSPERO (CRD42020179508). All references were imported in ‘Zotero’ reference manager.

### Study selection

For the purposes of this meta-analysis, we included studies reporting PCa detection rates among patients at risk of PCa according to their clinical parameters, of IB-TB, or FUS-TB, or COG-TB alongside TRUSGB. The exclusion criteria were: inclusion of patients with previously diagnosed PCa, on active surveillance, or mixed populations where this group were not reported on separately. We also excluded studies if data for patients biopsy status was not reported, or, if a previous negative biopsy population was included, we excluded studies where data for with the population no or at least one negative prior biopsy were not separately reported upon. Finally, we excluded studies if the MRI acquisition was not in accordance with the 2012 ESUR guidelines [7], or if studies used alternative targeted biopsy strategies such as multiparametric ultrasound.

### Quality assessment

The methodological quality of the studies was evaluated using the Quality Assessment of Diagnostic Accuracy Studies-2 (QUADAS-2) tool [8]. The assessment was performed by a single reviewer (AP) and checked by a second (HUA). QUADAS-2 is a tool recommended for use in systematic reviews to evaluate the risk of bias and the applicability of primary diagnostic accuracy studies [8].

### Data extraction

The initial data extraction was performed by a single reviewer (AP) in accordance with the START recommendations [9] and double-checked by a second (HUA). Data collected were: the recruitment method (clinical parameters); investigated population characteristics and sample size; the method of MRI acquisition and evaluation; MRI findings and/or PI-RADS score; the threshold for MRI positivity; biopsy method; whether the comparison between these two methods was made; the definition of csPCa and finally the detection rates of csPCa, and clinically insignificant PCa per patient and per core, where available.

### Data analysis

The analysis was conducted to answer our three stated aims. For this, we first focused only on studies that reported detection rates with both MRI-GB and TRUS-GB. Therefore, the final sample size of the study population refers to the number of patients that underwent both techniques. We calculated the effect sizes with their corresponding measures of variation prior to performing the meta-analysis.

We combined the data of all studies that reported employing any type of MRI-GB and compared it with TRUS-GB. It was obligatory that the studies reported results of both TRUS-GB and MRI-GB separately. The main accuracy measure – cancer detection rate (CDR) - was calculated by dividing the number of patients with detected cancer by TRUS-GB (or MRI-GB), with the total number of patients who were detected with cancer by the combination of TRUS-GB and MRI-GB. Relative CDR was expressed as the relative ratio between the CDR of MRI-GB and TRUS-GB. The standard error of each of the accuracy statistics was obtained by employing the formula for the standard error of a relative risk without taking the paired nature into account because not all studies reported their data in a paired format [10].

Superiority of clinically insignificant PCa detection was determined by comparison of the diagnostic yield (the likelihood that the biopsy procedure provides such a diagnosis) of each approach. In this respect, a lower number is advantageous and therefore the technique was regarded as ‘superior’. The yield of detecting clinically insignificant PCa (defined by each included study) was calculated by dividing the number of patients with insignificant PCa (the number of patients with any PCa minus the number of patients with csPCa) with the total number of patients that underwent biopsy. The relative yields were calculated by dividing the yield of MRI-GB with the yield of TRUS-GB. We pooled the estimates by conducting random-effects meta-analysis on precalculated effect sizes with metagen function of the meta package (R studio Version1.2.1335), using the generic inverse variance method and Sidik-Jonkman as a between-study-variance estimator. We assessed the between-study heterogeneity with Cochrane’s Q and I^2^ tests [11, 12]. I^2 ^> 50% with *p* < 0.05 indicates significant heterogeneity. All analyses were done for csPCa and insignificant PCa detection rates. Publication bias was explored by inspecting the funnel plot and by employing Eggar’s test.

To answer the third review question, we used the accuracy measurements we defined above and focused on studies that reported one of the MRI-GB techniques (IB-TB or FUS-TB or COG-TB). The within-study variances were calculated based on the exact binomial distribution. The differences in sensitivity of the three techniques was compared by conducting the mixed subgroup analysis with the use of subgroup.analysis.mixed.effects function (dmetar package).

We conducted two additional subgroup analyses––one based on the methodological quality and the other based on prior biopsy history. The former pooled the effect sizes only from the studies with low risk of bias and low concerns regarding applicability. The latter included three groups (biopsy naive, negative biopsy and a mixed group of both types of patients). We additionally performed a focused analysis that excluded the mixed population group. These analyses were performed for csPCa and clinically insignificant PCa detection rates. The extracted data were computed and pre-calculated in Microsoft Excel, version 2010, while the meta-analyses were performed in R studio Version 1.2.1335 (Boston, MA, USA).

## Results

### Search and selection

**Fig. 1:**
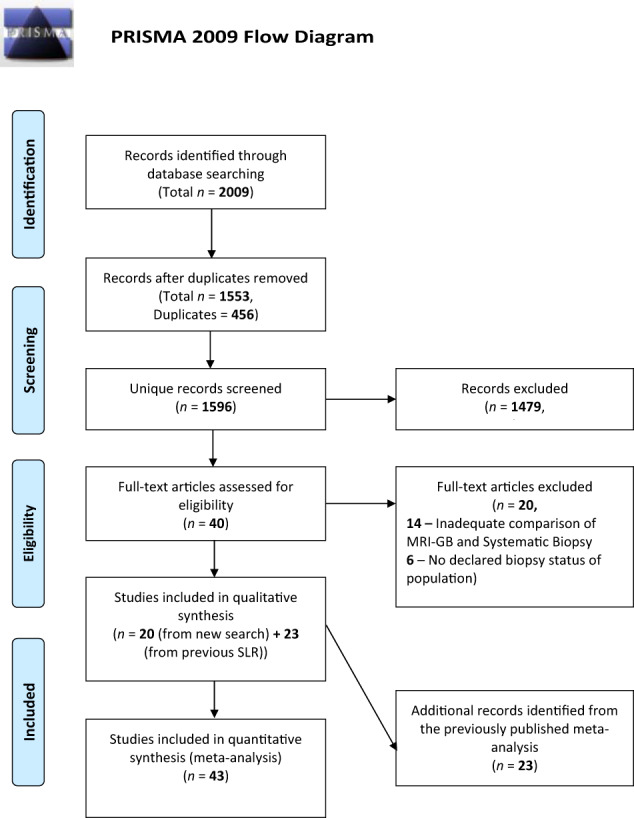
PRISMA Flow Diagram. Here we demonstrate the study selection process for the study. Ultimately, 43 studies were included in teh meta-analysis.

The systematic search of the three databases yielded a total of 1553 articles after removing 456 duplicates. We identified 40 new studies that assessed the diagnostic accuracy of some of the three MRI-GB techniques. Out of the identified 40 studies, 14 either did not compare the PCa detection rates between MRI-GB and TRUS-GB, or did not perform these comparisons in the same population of patients, thus they were not included in the meta-analysis. Of the remaining 26 studies, six did not report any information about the biopsy status of the study population, leaving 20 studies that were merged with the studies from the systematic review by Wegelin et al. [5]. The previous systematic review included 23/43 studies included in their meta-analysis [5] Merging new studies with those 23 from the previously published meta-analysis [5] provided a total of 43, including 8456 men, were finally included in our meta-analysis (Supplementary Table 1). The complete selection process of the studies is presented in the PRISMA flow chart (Fig. 1). All 20 newly identified studies, containing an additional 4675 men, were included in the meta-analysis comparing the three MRI-GB techniques.

### Quality assessment

Quality was evaluated only in studies included in the meta-analysis (*n* = 43) (Supplementary Table 1). All studies were estimated to have low risk regarding applicability to the current review (Supplementary figure 1b). Twenty-five studies were deemed to have a high risk of selection bias [13–55] (Supplementary figure 1a).

### Population

We observed high variability of study population size, characteristics and mean PSA level in the studies included in the meta-analysis. The sample size varied between 20 and 1003 patients, who were aged between 59.7 and 70 years, who either had no or at least one prior negative biopsy. The mean PSA level ranged from 5.7 to 11.0 ng/ml. Most studies involved a patient group with PSA < 10 ng/ml (*n* = 38). A 3-Tesla scanner was used in 77% of studies (*n* = 33). The most commonly used MRI-GB technique was FUS-TB (*n* = 31), followed by COG-TB (*n* = 8) and then IB-TB (*n* = 5). The definition of the threshold for performing targeted biopsy differed between studies. The PI-RADS classification system was the most frequent (*n* = 34), and a score of >/= 3 was the most used cutoff. The studies varied considerably in the definition of csPCa as well; therefore we performed the analysis depending on the definition given in the original articles.

### MRI-GB versus TRUS-GB

#### Does MRI-GB result in a higher CDR for clinically significant PCa compared with TRUS-GB?

**Fig. 2:**
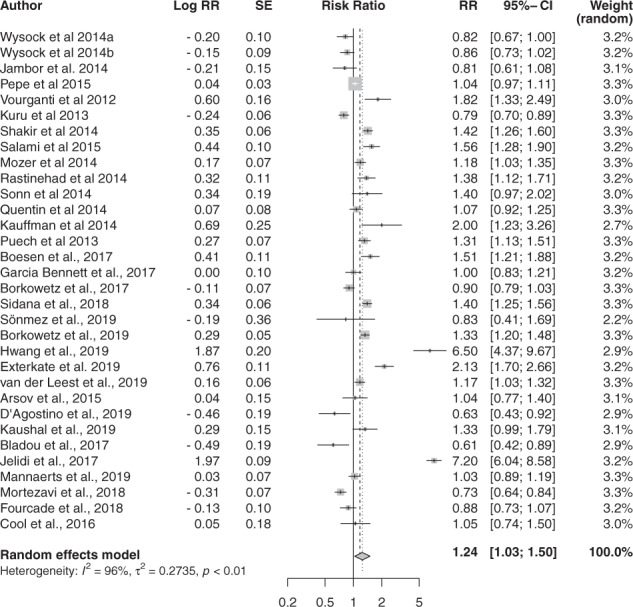
Cancer detection rate for csPCa. Forest plot representing relative CDR of MRI-GB to TRUS-GB.

We observed a statistically significant difference between the sensitivity of MRI-GB and TRUS-GB techniques for csPCa, with a pooled relative CDR of 1.24 [95% CI 1.03; 1.50, *p* = 0.02] although with significant heterogeneity among the studies (I^2 ^= 95.9%) (Fig. 2). The pooled CDR for MRI-GB was 0.83 [95% CI 0.76–0.90] and for TRUS-GB 0.63 [95% CI 0.53–0.74]. Heterogeneity was highly significant––83.7% for MRI-GB and 97.7% for TRUS-GB. We observed no significant evidence of publication bias (*p* = 0.39, Supplementary Figure 2).

#### Does MRI-GB result in a lower CDR for insignificant PCa compared with TRUS-GB?

**Fig. 3:**
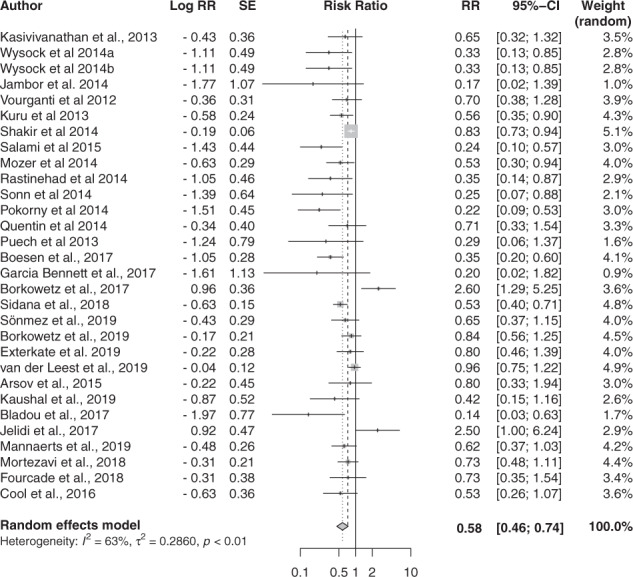
Relative yield for insignificant PCa detection. Forest plot representing the relative yield for detecting insignificant PCa of MRI-GB to TRUS-GB.

MRI-GB was less likely to result in a diagnosis of clinically insignificant PCa. The diagnostic yield for MRI-GB was 0.08 [95% CI 0.06; 0.11] (I^2 ^= 93.1%) and the yield for TRUS-GB was 0.15 [95% CI 0.12; 0.17] (I^2 ^= 92.7%). The pooled analysis showed that MRI-GB performed significantly better (*p* < 0.0001) with a pooled relative yield of 0.58 [0.46; 0.74], and moderate heterogeneity between the studies of 63.3% (Fig. 3). There was significant publication bias among the studies (*p* = 0.003, Supplementary Figure 3).

#### Which MRI-GB technique performed best at detecting csPCa and avoiding detection of insignificant PCa?

We observed no statistically significant differences between the three MRI-GB techniques in detecting csPCa (*p* = 0.55) with IB-TB showing a pooled CDR of 0.87 [95% CI 0.81–0.93], COG-TB 0.81 [95% CI 0.69–1.03] and FUS-TB 0.81 [95% CI 0.73-0.91]. There was no statistically significant difference between the three techniques in detecting clinically-insignificant PCa (*p* = 0.46); IB-TB had a pooled yield of 0.10 [95% CI 0.03–0.31]; COG-TB 0.05 [95% CI 0.02–0.11], and FUS-TB 0.08 [95% CI 0.06–0.11].

### Subgroup analysis

Due to the high heterogeneity among the included studies, we performed a subgroup analysis including only studies with low risk of bias and low concerns of applicability (*n* = 18). This revealed a relative pooled CDR of 1.23 (1.02; 1.47) in the detection of csPCa in favour of MRI-GB (*n* = 11). The heterogeneity was high (I^2 ^= 90.4%).

The analysis also found the pooled relative CDR in detecting csPCa trended towards higher in patients that had at least one prior negative biopsy (1.59 [95% CI 1.16-2.16]) compared to biopsy-naive men (1.06 [95% CI 0.94–1.20]) although this did not reach statistical significance (*p* = 0.06).

## Discussion

In summary, we first found a significant difference in CDR between TRUS-GB and MRI-GB for the detection of csPCa. We also observed considerably higher values of relative CDR after updating the analysis with newly identified studies than those in the previously published systematic review [5]). Third, MRI-GB detected fewer clinically insignificant cancers. Fourth, there was no statistically significant difference between the three techniques in CDR for csPCa.

Whilst the replacement of TRUS-GB with MRI-GB is technically achievable, the removal of systematic biopsy from our diagnostic pathway is controversial. This is due to concerns over missing significant disease ‘hidden’ in areas of the prostate that appear normal on MRI. Indeed, this analysis suggests that whilst superior, an MRI-GB approach alone would miss 17% of cases of csPCa. However, by comparison, TRUS-GB would miss 37% of such cases. This is an expected finding. For example, missing 40% of csPCA is broadly in keeping with the literature [1]. The multicentre PRECISION trial demonstrated the superiority of MRI-GB over TRUS-GB in terms of both detection of csPCa and of overdiagnosis of clinically insignificant PCa [56]. However, some csPCa that is missed by TRUS-GB is detected by MRI-GB and vice versa [57]. A combined approach of both systematic and targeted biopsy may be optimal, although these ‘missed’ cancers are likely to be low volume, low grade or with small amounts of pattern 4, leading to ongoing controversy as to their clinical importance [58].

The debate over which targeting approach is more accurate is ongoing. None of the aforementioned approaches has proven its superiority in a direct comparative trial. In our analysis, we did not find any approach superior in the critical aspect of detecting csPCa. A recently published RCT directly comparing all three approaches found no difference in csPCa detection rates, although the study was powered for the detection of any cancer [59]. The literature may be limited on this topic with highly expert practitioners conducting the COG-TB from which results are reported. Drawing strong conclusions with regard to the presence or absence of superiority of any technique is also challenging due to the significant heterogeneity between included studies. We also found evidence of publication bias in the studies reporting detection rates of csPCa and clinically insignificant disease.

Our meta-analysis has some limitations. First, there was significant patient selection bias in the included studies as in general only men with suspicious MRIs were included. This does not reflect a biopsy-naive population, arguably the most clinically relevant patient group to which this effect applies. If men with non-suspicious MRIs were included, it is likely the effect would be larger [57]. It should therefore be kept in mind that these studies all evaluate the efficacy of an mpMRI scan *and* a biopsy strategy.

Second, there was significant heterogeneity in the included studies. Some variation was observed in MRI acquisition with variance in scanner resolution, the threshold for biopsy and most importantly, there was inconsistency in the MRI reporting standards. However, all the studies included in the meta-analysis followed the European Society of Genito-Urinary (ESUR) prostate mpMRI guideline standards [7] and 34 of 43 studies used PIRADS as their chosen reporting system. Further, there was variance in the indications for biopsy. As we have already stated, a biopsy-naive population is most relevant and the least biased. We included studies with the multitude of indications that physicians face in clinical practice in order to improve our findings’ generalizability for the reader. Naturally, a more homogeneous group of studies is ideal. However, if only studies with biopsy-naive populations were included only 13 studies with 1878 patients could be included; if only studies using PIRADS with a cutoff score of > /=3 were included in addition to a biopsy-naïve population then only two studies could be included in the meta-analysis.

Third, the thresholds used for csPCa varied although most used the presence of any Gleason >/= 3 + 4 when it was reported. Further, and more problematic, is that commonly used definitions of csPCa are modelled from systematic biopsy data and as such their utility in targeted biopsy has inherent challenges. For example, targeted biopsies by design take multiple cores from a single area of interest. Thus, when total cancer core lengths or numbers/percentages of positive cores are used in this setting there is a significant risk of over-estimation of risk; something we have described as the potential Will–Rogers phenomenon [60].

Fourth, given the aforementioned drawbacks of TRUS-GB [1] we acknowledge that its use as a reference standard for determining the ideal MRI-GB technique is not optimal. Template mapping biopsies would represent a better reference standard for answering our third question. However, one should bear in mind that a recent RCT also found no advantage to any technique [59].

Finally, there were variations in biopsy technique between studies. For example, by the number of cores taken, or whether TRUS-GB or MRI-GB was performed first. If the former is true then prostatic swelling may affect diagnostic accuracy due to the swelling altering prostatic morphology. There are also numerous FUS-TB systems available on the market. Some use rigid fusion systems where the MR-images simply overlay the USS images, and some use elastic fusion systems where double contouring accounts for prostatic deformation by the ultrasound probe. A definitive, multi-user, multi-centre, multi-fusion platform randomized controlled trial sufficiently powered for csPCa to overcome these incorporation biases is needed.

## Conclusions

In men where PCa is suspected on MRI, MRI-GB offers superior rates of csPCa detection and significantly reduces diagnoses of insignificant PCa compared to TRUS-GB. In terms of the superiority of MRI-GB techniques we found no significant differences in CDR for csPCa. More than half of the included studies were subject to significant selection bias and thus conclusions must be tempered. Further, in studies reporting on COG-TB, highly expert practitioners performed the biopsies, possibly overstating the sensitivity of the technique. A robust RCT design appropriately powered for csPCa as the primary outcome measure is required to provide a definitive answer.

## Supplementary information

Supplementary Figure Legends

Supplementary Table 1

Supplementary Figure 1a

Supplementary Figure 1b

Supplementary Figure 2

Supplementary Figure 3

## Data Availability

Example r code used for this analysis is found in a statistical methodology paper by Shim et al. [61]
