# Supplementary material for: Diagnostic accuracy of magnetic resonance imaging targeted biopsy techniques compared to transrectal ultrasound guided biopsy of the prostate: a systematic review and meta-analysis
Source: Prostate Cancer Prostatic Dis. 2021 Sep 21;25(2):174–9. doi: 10.1038/s41391-021-00449-7 (PMC9184263; doi:10.1038/s41391-021-00449-7)
Supplement: Supplementary file 1 — Supplementary Figure Legends [file 41391_2021_449_MOESM1_ESM.docx]

**Supplementary Figure Legends**

Supplementary figures 1a & b: Summary tables of the QUADAS-2 bias assessment.

Supplementary figure 2: Funnel plot demonstrating no significant publication bias was found in studies reporting CDR for csPCa (Eggar’s test p=0.39)

Supplementary figure 3: Funnel plot demonstrating significant publication bias was found in studies reporting CDR for insignificant PCa (Eggar’s test p=0.003)
