# Supplementary material for: Diagnostic accuracy of magnetic resonance imaging targeted biopsy techniques compared to transrectal ultrasound guided biopsy of the prostate: a systematic review and meta-analysis
Source: Prostate Cancer Prostatic Dis. 2021 Sep 21;25(2):174–9. doi: 10.1038/s41391-021-00449-7 (PMC9184263; doi:10.1038/s41391-021-00449-7)
Supplement: Supplementary file 2 — Supplementary Table 1 [file 41391_2021_449_MOESM2_ESM.docx]

| **Author, Year [Ref.]** | **Population** | **Recruitment criteria** | **No. of patients** | **Mean/ Median age (yr)** | **Mean/Median PSA (ng/ml)** | **MRI used; magnet strength** | **Coil used** | **Threshold for target** | **Target; approach** | **Systematic cores** | **Definition of clinically significant PCa** |
| --- | --- | --- | --- | --- | --- | --- | --- | --- | --- | --- | --- |
| MIYAGAWA et al 2010 **[14]** | Negative previous biopsy | Elevated PSA AND suspicious MRI | 85 | 69 | 9.9 | Philips Interna Pulsar 1.5T | PPA | None defined | MRI/TRUS fusion; transperineal | Yes | None defined |
| PARK et al 2011 **[15]** | Biopsy naïve | Elevated PSA and/or abnormal DRE | 44 | 63 | 6.1 | Philips Achieva 3T | PPA | Suspicious MRI; none defined | Cognitive TRUS; transrectal | Yes | None defined |
| PORTALEZ et al 2012 **[16]** | Negative previous biopsy | Elevated PSA AND/OR abnormal DRE AND suspicious MRI | 129 | 64.7 | 9.6 | Philips Achieva 1.5T; Siemens Avanto 1.5T | PPA | Regardless of MRI findings  (PIRADS) | MRI/TRUS fusion; transrectal | Yes | None defined |
| VOURGANTI et al 2012 **[17]** | Negative previous biopsy | Elevated PSA AND/OR abnormal DRE | 195 | 62 | 9.1 | Philips Achieva 3T | ERC & PPA | Regardless of MRI findings  >/= Low | MRI/TRUS fusion; transrectal | Yes | Gleason >/=3+4 |
| DELONGSHAMPS et al 2013 **[18]** | Biopsy naïve | Elevated PSA AND/OR abnormal DRE | 391 | 63.9 | 8.5 | Unknown 1.5T | ERC & PPA | Sum score of ≥4 and ≥6 | Cognitive TRUS and MRI/TRUS fusion; transrectal | Yes | Gleason score 3+3 AND MCCL <5 mm AND single core positive |
| FIARD et al 2013 **[19]** | Negative previous biopsy OR Biopsy naïve | Elevated PSA AND/OR abnormal DRE | 30 | 64 | 6.3 | Philips Achieva 3T | PPA | PIRADS >/=5 | MRI/TRUS fusion; transrectal | Yes | d'Amico classification (Intermediate / High risk) or Gleason >/=3 |
| BORKOWETZ et al 2014 **[20]** | Mixed population | Elevated PSA AND/OR abnormal DRE AND MRI | 263 | 66 | 8.3 | Siemens Magnetom Verio 3T | PPA | PIRADS >/=2 | MRI/TRUS fusion; transrectal | Yes | Gleason >/=3+4 |
| KURU et al 2013 **[21]** | Negative previous biopsy OR Biopsy naïve | Elevated PSA AND/OR abnormal DRE | 347 | 65.3 | 9.9 | Siemens Magnetom Trio 3T | PPA | Regardless of MRI findings | MRI/TRUS fusion; transperineal | Yes | NCCN criteria (intermediate and high risk) |
| PUECH et al 2013 **[22]** | Negative previous biopsy OR Biopsy naïve | Elevated PSA AND/OR abnormal DRE | 95 | 65 | 10.1 | Philips Gyroscan Intera 1.5T; Siemens Symphony 1.5T | PPA | PIRADS >/=3 | Cognitive / MRI TRUS fusion | Yes | Gleason >/=3+4; Gleason 3+3 AND MCCL >3mm |
| WYSOCK et al 2014 **[23]** | Mixed population | Elevated PSA AND/OR abnormal DRE AND suspicious MRI | 67 | 65 | 5.1 | Siemens Magnetom Trio 3T | PPA | PIRADS >/=2 | Cognitive TRUS AND MRI/TRUS fusion; transrectal | Yes | Gleason score >/=3+4 |
| SALAMI et al 2014 **[24]** | Negative previous biopsy OR Biopsy naïve | Elevated PSA AND/OR abnormal DRE AND suspicious MRI | 140 | 65.8 | 9 | Siemens Magnetom Verio 3T | ERC & PPA | PIRADS >/=2 | MRI/TRUS fusion; transrectal | Yes | Epstein criteria; Gleason >/=3+4; MRI lesion volume >0.2mL |
| IWAMOTO et al 2014 **[25]** | Biopsy naïve | Elevated PSA | 238 | 69.2 | 9.6 | Philips Achieva 1.5T; Siemens Magnetom Skyra 3T | Unknown | Suspicious MRI; none defined | Cognitive TRUS; transrectal | Yes | Gleason score >/=3+4 |
| POKORNY et al 2014 **[26]** | Biopsy naïve | Elevated PSA AND/OR abnormal DRE | 142 | 63 | 5.6 | Siemens Magnetom Skyra 3T | PPA | PIRADS >/=3 | Cognitive TRUS and MRI/TRUS fusion; transperineal | Yes | Gleason 3+3 AND MCCL >/=6mm; Gleason 3+4 AND MCCL >/= 4mm; Gleason >/=4+3 |
| QUENTIN et al 2014 **[27]** | Biopsy naïve | Elevated PSA | 128 | 66.1 | 6.7 | Siemens Magnetom Trio 3T | PPA | None defined  (PIRADS) | In-bore MRI; transrectal | Yes | Gleason >/=3=4; Gleason 3+3 AND TCCL >5mm |
| RASTINEHAD et al 2014 **[28]** | Negative previous biopsy OR Biopsy naïve | Elevated PSA AND/OR abnormal DRE AND MRI | 105 | 65.8 | 9.2 | Siemens Magnetom Verio 3T | ERC & PPA | >/= Low risk (NIH criteria) | MRI/TRUS fusion; transrectal | Yes | Epstein criteria; Gleason >/=3+4; MRI lesion volume >0.2mL |
| SHAKIR et al 2014 **[29]** | Negative previous biopsy OR Biopsy naïve | Elevated PSA AND/OR abnormal DRE AND suspicious MRI | 1003 | 62.1 | 6.7 | Philips Achieva 3T | ERC & PPA | Suspicious MRI; no threshold defined | MRI/TRUS fusion; transrectal | Yes | Gleason score >/=4+3 |
| SONN et al 2014 **[30]** | Negative previous biopsy | Elevated PSA | 105 | 65 | 7.5 | Siemens Magnetom Trio 3T | PPA | PIRADS >/=2 | MRI/TRUS fusion; transrectal | Yes | Gleason >/=3+4 OR 3+3 AND MCCL >/=4mm |
| KAUFMAN et al 2015 **[31]** | Negative previous biopsy | Elevated PSA AND suspicious MRI | 35 | 68 | 9.4 | Siemens Magnetom Espree 1.5T | ERC | Regardless of MRI findings  (PIRADS) | In-bore MRI; transrectal | Yes | d’Amico classification (intermediate and high risk) and Epstein criteria |
| JAMBOR et al 2015 **[32]** | Biopsy naïve | Elevated PSA | 53 | 66 | 7.4 | Siemens Magnetom Verio 3T | PPA | PIRADS >/=4 | Cognitive TRUS; transrectal | Yes | Gleason >/=3+4; Gleason 3+3 AND MCCL >/= 3mm |
| BOESEN et al 2015 **[33]** | Negative previous biopsy | Elevated PSA AND/OR abnormal DRE | 83 | 63 | 11 | Philips Achieva 3T | PPA | None defined  (PIRADS) | Cognitive TRUS; transrectal | Yes | Epstein criteria |
| SALAMI et al 2015 **[34]** | Negative previous biopsy | Elevated PSA AND/OR abnormal DRE AND suspicious MRI | 140 | 65.8 | 9 | Siemens Magnetom Verio 3T | ERC & PPA | PIRADS >/=2 | MRI/TRUS fusion; transrectal | Yes | Epstein criteria; Gleason >/=3+4; MRI lesion volume >0.2mL |
| SHOJI et al 2015 **[54]** | Biopsy naïve | Elevated PSA AND/OR abnormal DRE | 20 | 70 | 7.4 | GE Signa 1.5T | PPA | PIRADS >/=2 | MRI/TRUS fusion; transperineal | Yes | Gleason >/=3+4; MCCL >4mm |
| MOZER et al 2015 **[36]** | Biopsy naïve | Elevated PSA AND suspicious MRI | 152 | 63 | 6 | Philips Achieva 1.5T | PPA | PIRADS >/=2 | MRI/TRUS fusion; transrectal | Yes | Gleason >/=3+4; Gleason 3+3 AND MCCL >/= 4mm |
| PEPE et al 2015 **[37]** | Negative previous biopsy | Elevated PSA | 100 | 64 | 8.6 | Philips Achieva 3T | PPA | PIRADS >/=4 | Cognitive TRUS; transperineal | Yes | Gleason >/=3+4; Gleason 3+3 AND TCCL >50% |
| ARSOV et al 2015 **[38]** | Negative previous biopsy | Elevated PSA AND/OR abnormal DRE | 210 | Arm A: 66, Arm B: 68 | Arm A: 10.0; Arm B: 10.8 | Siemens Magnetom Trio 3T | PPA | None defined  (PIRADS) | Arm A: IB-TB; FUS-TB + TRUS-GB | Yes | Gleason >/=3+4 |
| CASH et al 2016 **[39]** | Negative previous biopsy OR Biopsy naïve | Elevated PSA AND/OR abnormal DRE | 408 | 67 | 9.3 | Siemens Magnetom Skyra 3T | PPA | PIRADS >/=2 | MRI/TRUS fusion; transrectal | Yes | Gleason >/=3+4 OR 3+3 AND MCCL >/=4mm |
| MARIOTTI et al 2016 **[40]** | Mixed population | Elevated PSA AND/OR rise in PSA velocity AND/OR abnormal DRE | 389 | 62.8 | 8 | Philips Achieva 3T; Philips Ingenia 3T; Siemens Magnetom Trio 3T | ERC & PPA | Likert >/=3 | MRI/TRUS fusion; transrectal | Yes | Gleason 3+4 AND >/=50% TCCL; Gleason >/=4+3 |
| COOL et al 2016 **[41]** | Previous biopsy with ASAP OR Biopsy naïve | Elevated PSA AND/OR abnormal DRE | 100 | 59.4 | 6 | GE Unknown 3T | ERC & PPA | PIRADS >/=2 | MRI/TRUS fusion; transrectal | Yes | Gleason >/=3+4; Gleason >/=4+3 OR MCCL >6mm |
| BOESEN et al 2017 **[42]** | Negative previous biopsy | Elevated PSA AND/OR abnormal DRE AND/OR suspicious TRUS images | 206 | 65 | 12.8 | Philips Ingenia 3T | PPA | PIRADS >/=2 | MRI/TRUS fusion; transrectal | Yes | Gleason >/=3+4 |
| GARCIA BENNETT et al 2017 **[43]** | Biopsy naïve | Elevated PSA AND/OR elevated PSA density AND/OR elevated PSA velocity AND/OR abnormal DRE | 60 | 64.1 | 7.2 | GE Signa 3T | PPA | PIRADS >/=4 | MRI/TRUS fusion; transrectal | Yes | Gleason >3+3 OR MCCL >3mm |
| BORKOWETZ et al 2017 **[44]** | Biopsy naïve | Elevated PSA AND/OR abnormal DRE | 214 | 63 | 6.2 | Siemens unknown 3T | PPA | PIRADS >/=2 | MRI/TRUS fusion; transperineal | Yes | Gleason score >/=3+4 |
| BLADOU et al 2017 **[45]** | Negative previous biopsy OR Biopsy naïve | Elevated PSA AND/OR abnormal DRE | 203 | 66 | 7.9 | Unknown 3T | PPA | Likert >/=2 | MRI/TRUS fusion; transrectal | Yes | Gleason score >/=3+4 OR Lesion volume >/= 0.5mL |
| JELIDI et al 2017 **[46]** | Negative previous biopsy OR Biopsy naïve | Elevated PSA AND suspicious MRI | 130 | 62.9 | 9.5 | Philips Achieva 3T | ERC & PPA | Suspicious MRI; no threshold defined  (PIRADS) | MRI/TRUS fusion; transrectal | Yes | Gleason >/=3+4; Gleason 3+3 AND MCCL >5mm |
| SIDANA et al 2018 **[47]** | Negative previous biopsy | Elevated PSA AND suspicious MRI | 799 | 63.1 | 8.5 | Unknown | Unknown | PIRADS >/=3 | MRI/TRUS fusion; transrectal | Yes | Gleason >/=3+4 |
| MORTEZAVI et al 2018 **[48]** | Negative OR positive previous biopsy OR Biopsy naïve | Treatment naïve | 415 | 64 | 6.7 | Siemens Magnetom Skyra 3T | ERC & PPA OR PPA | PIRADS >/=3 | MRI/TRUS fusion; transperineal | Yes | Gleason >/=3+4; Gleason 3+4 AND MCCL >/=4mm; Gleason >/=4+3 OR MCCL >/=6mm; Gleason >/=3+4 OR MCCL >/=4mm |
| FOURCADE et al 2018 **[49]** | Negative previous biopsy OR Biopsy naïve | Elevated PSA AND/OR abnormal DRE | 191 | 66 | 9 | Philips Achieva 3T | PPA | PIRADS >/=3 | MRI/TRUS fusion; transrectal | Yes | Gleason >/=3+4; MCCL >/=4mm |
| SONMEZ et al 2019 **[50]** | Biopsy naïve | Elevated PSA AND/OR abnormal DRE | 80 | 61 | 5.91 | Siemens Magnetom 1.5T | Unknown | PIRADS >/=3 | MRI/TRUS fusion; transrectal | Yes | ISUP criteria |
| HWANG et al 2019 **[51]** | Negative previous biopsy | Elevated PSA | 67 | 66 | 5.7 | Philips Achieva 3T; Philips Ingenia 3T | PPA | PIRADS >/=3 | MRI/TRUS fusion; transrectal | Yes | Gleason >/=4+3 in single core; Gleason 3+3 OR 3+4 in two or more cores |
| EXTERKATE et al 2019 **[52]** | Negative previous biopsy | Elevated PSA AND/OR abnormal DRE | 152 | 66 | 11 | Unknown 3T | Unknown | PIRADS >/=3 | MRI/TRUS fusion; In-bore fusion; Cognitive fusion | Yes | Gleason >/=3+4 |
| VAN DER LEEST et al 2019 **[53]** | Biopsy naïve | Elevated PSA | 626 (317 TB AND SB biopsy) | 65 | 6.4 | Siemens Magnetom Skyra 3T | Unknown | PIRADS >/=3 | In-bore MRI; transrectal | Yes | Gleason >/=3+4; Gleason >/=4+3; Gleason >/=3+3(Large) |
| D'AGOSTINO et al 2019 **[54]** | Negative previous biopsy OR Biopsy naïve | Elevated PSA AND/OR abnormal DRE | 39 | 65.5 | 6.6 | Philips Achieva 1.5T | PPA | PIRADS >/=3 | In-bore MRI; transrectal | Yes | ISUP criteria |
| KAUSHAL et al 2019 **[55]** | Negative previous biopsy OR Biopsy naïve | Elevated PSA AND/OR abnormal DRE | 131 | 63.5 | 9.75 | Philips Ingenia 3T | Unknown | PIRADS >/=3 | MRI/TRUS fusion; transrectal | Yes | Gleason score >/=4+3 |
| MANNAERTS et al 2019 **[56]** | Negative previous biopsy OR Biopsy naïve | Elevated PSA AND/OR abnormal DRE | 255 | 65 | 8.1 | Unknown 1.5T; Unknown 3T | Unknown | PIRADS >/=3 | MRI/TRUS fusion; transrectal | Yes | Gleason score >/=3+4 |

**Table 1:** Characteristics of patients included in the meta-analysis. Note—PSA = prostate-specific antigen level, DRE = digital rectal examination, T = Tesla, MRI = magnetic resonance imaging, PIRADS = prostate imaging-reporting and data system, PPA = phased array coil, ERC = endorectal coil, TRUS – transrectal ultrasound, ISUP = International Society of Urological Pathology, NCCN = National Comprehensive Cancer Network, ASAP = atypical small acinar proliferation
