# Supplementary figures and images for: Diagnostic accuracy of magnetic resonance imaging targeted biopsy techniques compared to transrectal ultrasound guided biopsy of the prostate: a systematic review and meta-analysis
Source: Prostate Cancer Prostatic Dis. 2021 Sep 21;25(2):174–9. doi: 10.1038/s41391-021-00449-7 (PMC9184263; doi:10.1038/s41391-021-00449-7)

## Risk of Bias

■ High ■ Low

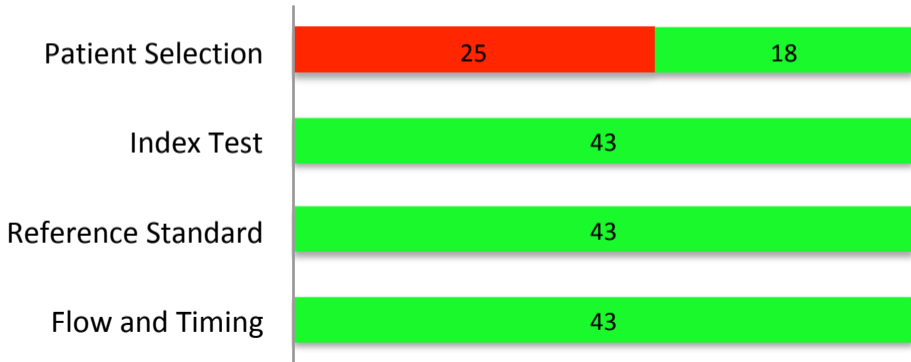

Supplement: Supplementary file 3 — Supplementary Figure 1a [file 41391_2021_449_MOESM3_ESM.pdf]

## Applicability Concerns

■ High ■ Low

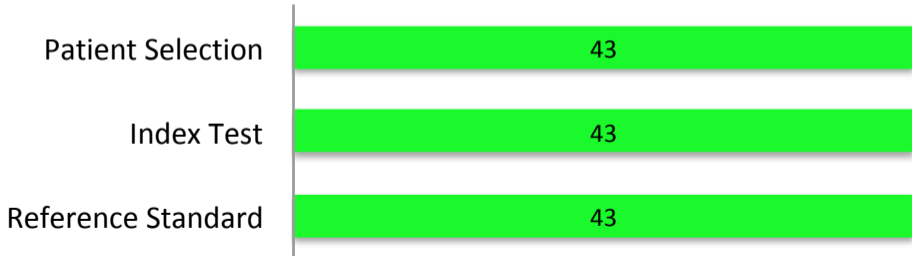

Supplement: Supplementary file 4 — Supplementary Figure 1b [file 41391_2021_449_MOESM4_ESM.pdf]

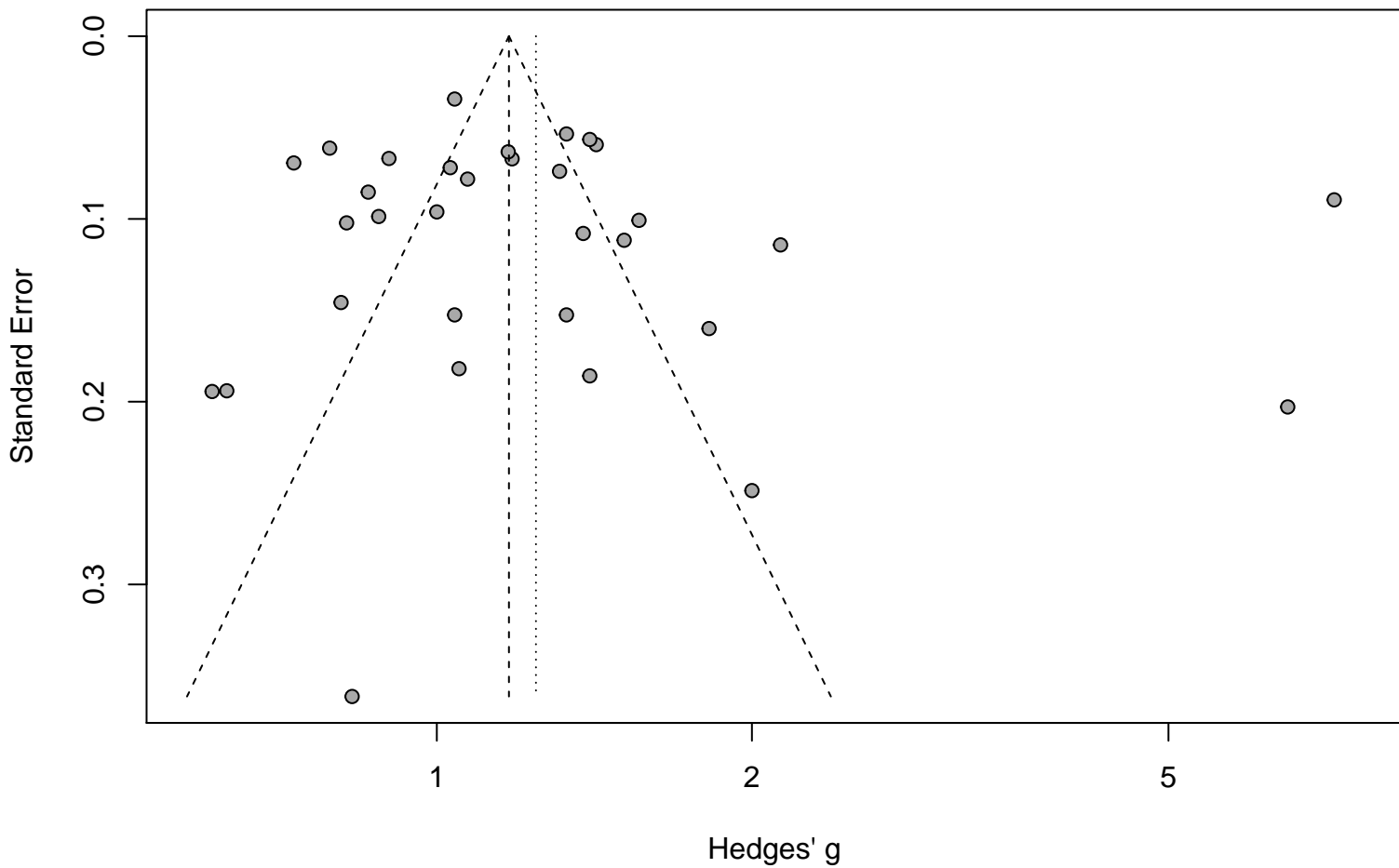

Supplement: Supplementary file 5 — Supplementary Figure 2 [file 41391_2021_449_MOESM5_ESM.pdf]

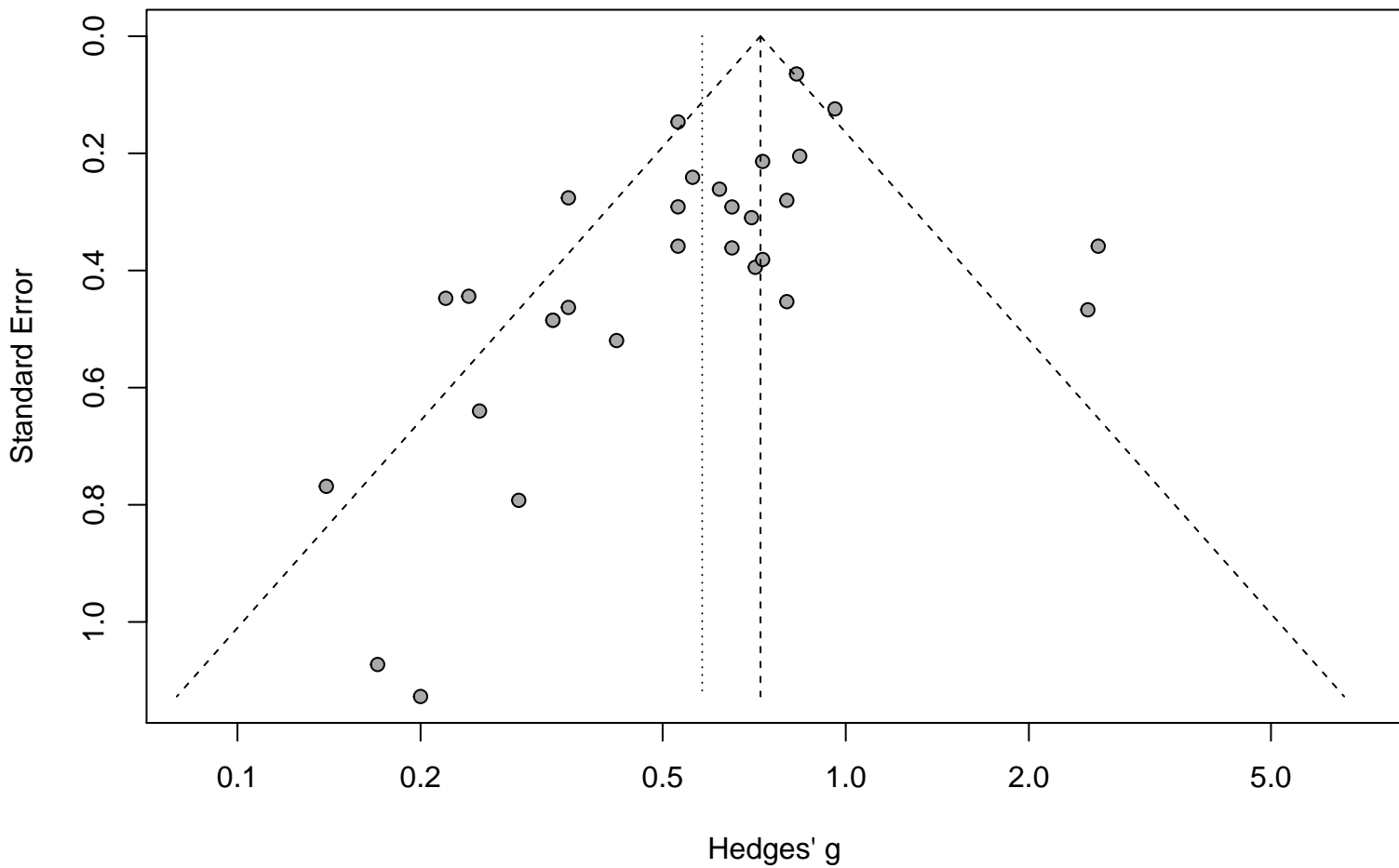

Supplement: Supplementary file 6 — Supplementary Figure 3 [file 41391_2021_449_MOESM6_ESM.pdf]
